# The Role of Serotonin during Skin Healing in Post-Thermal Injury

**DOI:** 10.3390/ijms19041034

**Published:** 2018-03-29

**Authors:** Alia Sadiq, Ahmed Shah, Marc G. Jeschke, Cassandra Belo, Muhammad Qasim Hayat, Sheeba Murad, Saeid Amini-Nik

**Affiliations:** 1 Faculty of Medicine, University of Toronto, Toronto, ON M5S 1A8, Canada; aliasadiq55@hotmail.com or alia.11.dirphd.56@asab.nust.edu.pk (Al.S.); ahm.shah@mail.utoronto.ca (Ah.S.); 2 Sunnybrook Research Institute, Toronto, ON M4N 3M5, Canada; cassandra.belo@gmail.com; 3 Atta-ur-Rahman School of Applied Biosciences (ASAB), National University of Sciences & Technology (NUST), H-12 Islamabad, Pakistan; mqasimhayat@hotmail.com (M.Q.H.); sheebamall@yahoo.com (S.M.); 4 Department of Surgery, University of Toronto, Toronto, ON M5T 1P5, Canada; 5 Molecular Immunology Unit, The Institute of Infection and Immunity, St. George’s, University of London, London SW17 0RE, UK; 6 Department of Laboratory Medicine and Pathobiology (LMP), University of Toronto, Toronto, ON M5S 1A8, Canada

**Keywords:** serotonin, fibroblasts, keratinocytes, migration, post thermal injury, wound healing

## Abstract

Post-burn trauma significantly raises tissue serotonin concentration at the initial stages of injury, which leads us to investigate its possible role in post burn wound healing. Therefore, we planned this study to examine the role of serotonin in wound healing through in vitro and in vivo models of burn injuries. Results from in vitro analysis revealed that serotonin decreased apoptosis and increased cell survival significantly in human fibroblasts and neonatal keratinocytes. Cellular proliferation also increased significantly in both cell types. Moreover, serotonin stimulation significantly accelerated the cell migration, resulting in narrowing of the scratch zone in human neonatal keratinocytes and fibroblasts cultures. Whereas, fluoxetine (a selective serotonin reuptake inhibitor) and ketanserin (serotonin receptor 2A inhibitor) reversed these effects. Scald burn mice model (20% total body surface area) showed that endogenous serotonin improved wound healing process in control group, whereas fluoxetine and ketanserin treatments (disruptors of endogenous serotonin stimulation), resulted in poor reepithelization, bigger wound size and high alpha smooth muscle actin (α-SMA) count. All of these signs refer a prolonged differentiation state, which ultimately exhibits poor wound healing outcomes. Collectively, data showed that the endogenous serotonin pathway contributes to regulating the skin wound healing process. Hence, the results of this study signify the importance of serotonin as a potential therapeutic candidate for enhancing skin healing in burn patients.

## 1. Introduction

Severe burn wound injuries have become a global public health challenge, which presents a significant financial, psychological and emotional burden on patients and physicians [1,2]. World Health Organization (WHO) estimates that fire-related burn injuries alone result in more than 300,000 deaths and loss of 10 million Disability Adjusted Life Years (DALYs) each year [3]. Although developing countries share most of the global burden of burn injuries but the mortality rate associated with burn injuries in developed countries such as United States (US) is still significant (5.3%) [4]. Clinically, high mortality in burn patients is mainly due to loss of skin cover resulting in increased metabolic demand, fluid loss and significant risk of infections [5,6,7]. Therefore, quick and permanent wound closure is necessary. Current treatments include grafting or reconstruction and skin engineering [8,9,10]; however, the ultimate therapeutic goal is to enhance and accelerate the wound healing process by activating respective signaling pathways of healing.

The attempt to regulate the wound healing process is based upon specific strategies to explore novel molecular pathways or key mediators of the healing process, such as the serotonergic system. The serotonergic system consists of serotonin and serotonergic receptor interactions to induce downstream signaling response. Serotonin or 5-hydroxytryptamine (5HT) is a monoamine neurotransmitter, well known for its antidepressant properties in the central nervous system (CNS). It induces various cellular responses through different serotonergic G-protein coupled receptors (GPCRs) which are further classified into seven classes of serotonin receptors (5HTR) 1 to 7 [11]. The key mediators of serotonergic effects outside the CNS are the platelets that store most of the peripheral serotonin in the form of dense granules [12]. Serotonin stimulation is known to play an important role in establishing hemostasis and tissue healing [13]. Serotonin’s regenerative and fibroproliferative potential has also been previously documented in different organ systems. For instance, blocking 5HTR signaling impairs liver regeneration mediated by platelet-derived serotonin [14]. Moreover, 5HTR2-mediated fibroproliferation has been identified in several organ systems, including the liver and the lung [15,16]. Interestingly, an increased endogenous skin serotonin level has been reported after cutaneous burns [17,18], but its significance has not been investigated yet. Literature search revealed that studies exploring the role of the serotonergic system in skin wound healing are limited in numbers, and particularly in the context of burn injuries.

A previous in vitro study suggests that serotonin promotes fibroblast proliferation in a dose-dependent manner [19]. Another earlier study documented that subcutaneous serotonin administration, in the uninjured skin, induced dermal fibroblast proliferation [20]. However, the effects of serotonin and serotonin inhibitors on fibroblasts during wound healing is yet to be explored [21]. To investigate these effects in the context of burns, is especially important because burn injuries result in dysregulated and prolonged inflammatory response leading to excessive fibroproliferation and hypertrophic scarring [22]. Thus, studying the effects of enhancement and inhibition of the serotonergic pathway on fibroblasts in burn injuries may have implication for both wound healing and hypertrophic scarring disorders.

Taking previous studies into account, the current study was based upon the hypothesis that serotonin signaling enhances fibroblast and keratinocyte proliferation and contributes to the healing mechanism. Up to the present time, there has been no experimental evidence to support this hypothesis. Since serotonin’s mechanism of action in skin wound healing is unknown, we designed this study to explore the effect of serotonin and its modulators including fluoxetine (selective serotonin reuptake inhibitors, SSRIs) and ketanserin (5HTR2a antagonist) during post burn wound healing. Primarily, the effect of the serotonergic pathway on fibroblasts and keratinocytes’ function was investigated (in vitro), because these cell types are critical to the process of restoring structural and functional integrity of skin during wound healing [23]. Later, we investigated the role of serotonin at the proliferative stage of burn wound healing (in vivo).

## 2. Results

### 2.1. Serotonin Enhances the Cell Survival and Proliferation (In Vitro Model)

**Figure 1:**
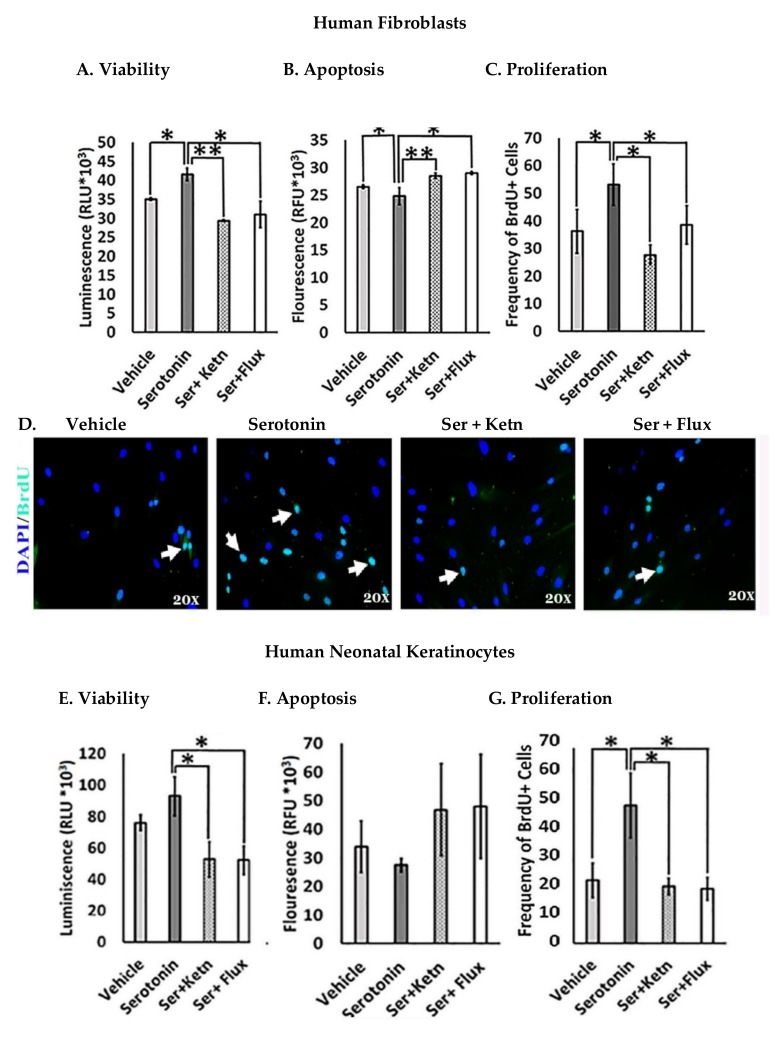

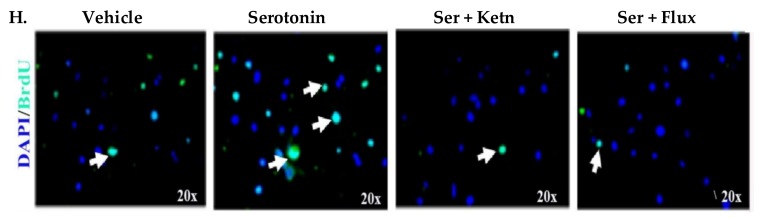
Comparative cellular effects of serotonin, ketanserin and fluoxetine drug treatments on (**A**,**E**) cell viability (**B**,**F**) apoptosis and (**C**,**G**) proliferation of human fibroblasts and neonatal keratinocytes cultures, respectively. (**D**,**H**) Immunofluorescent staining with anti-BrdU antibody (green) and 4, 6-diamidino-2 phenylindole (DAPI) nuclear stain (blue) in human fibroblasts and neonatal keratinocytes, respectively. White arrows marked BrdU + ve cells. Images scale; 20× resolution. Results were presented as mean ± Standard Deviation (SD), (* *p* < 0.05, ** *p* < 0.01), *n* = 5.

The effect of serotonin signaling on cell viability of human fibroblasts and neonatal keratinocytes was investigated (Figure 1). Results revealed that serotonin treatment enhanced cell survival significantly (Relative Luminescent Unit, RLU = 41.51 × 10^3^ ± 1.45) in human fibroblasts (Figure 1A) and keratinocytes (RLU = 98.9 × 10^3^ ± 4.6) (Figure 1E), as compared to the respective control group, whereas cell viability reduced significantly upon interrupted serotonin signaling by the addition of ketanserin (a 5HTR2a inhibitor) and fluoxetine. Results from apoptosis assay showed that serotonin induced significant anti-apoptotic effects (Relative Fluorescence Unit, RFU = 24.79 × 10^3^ ± 1.35) on fibroblasts (Figure 1B) but not in keratinocytes (Figure 1F). Moreover, cell proliferation is another cellular feature, which contributes to tissue replacement after injury [24]. The proliferative stage of wound healing is mainly characterized by propagation of fibroblasts and epidermal keratinocytes [25]. Therefore, experimental analysis of proliferation assay showed significantly increased proliferation in human fibroblast (BrdU + ve cells = 53.13 ± 6.6) (Figure 1C, D) and keratinocytes (BrdU + ve cells = 44.09 ± 2.4) (Figure 1G, H) upon serotonin treatment as compared to the respective control group. On the contrary, ketanserin and fluoxetine significantly inhibit proliferation in both cell types.

### 2.2. Serotonin Positively Regulates Cell Migration (In Vitro Model)

**Figure 2:**
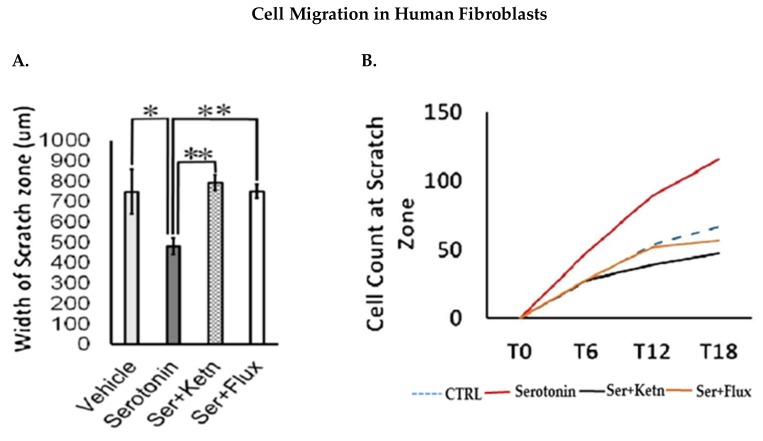

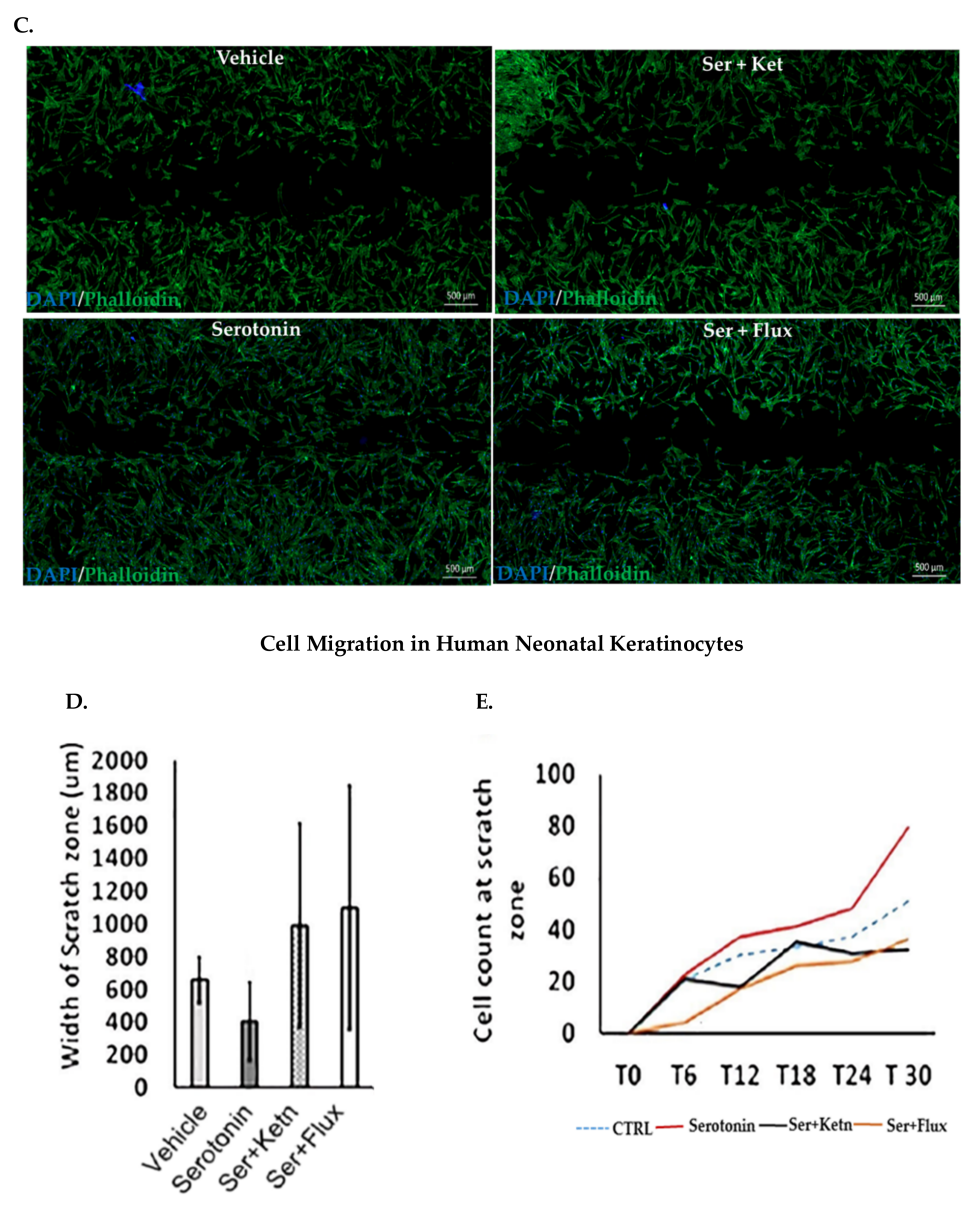

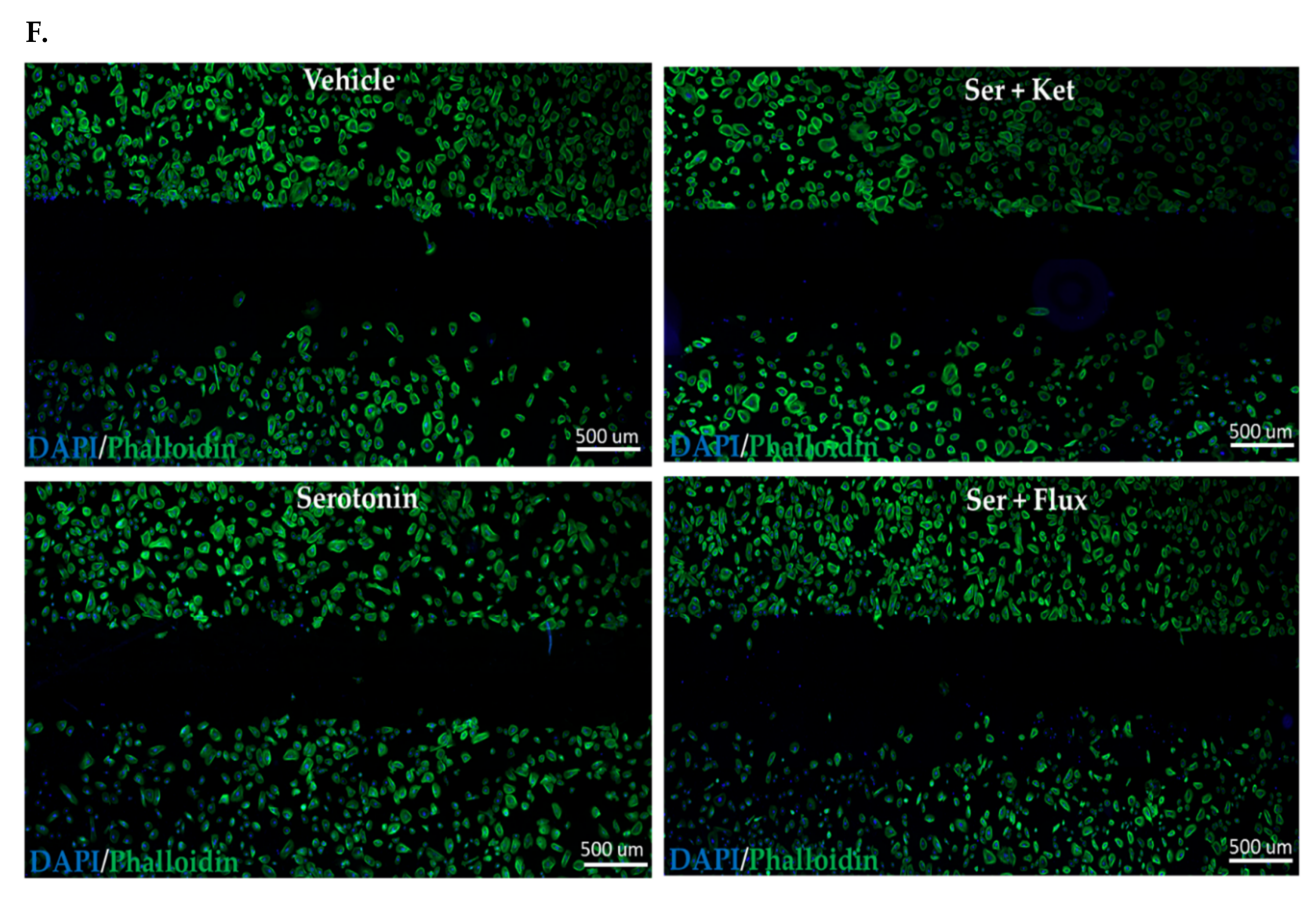
Comparative cellular effects of serotonin, ketanserin and fluoxetine drug treatments on cell migration, in human fibroblasts and neonatal keratinocytes cultures. (**A**) Migrating fibroblasts narrowed the scratch zone and width of scratch zone was measured in micrometers (μm) at Time 18 h. (**B**) Migrated fibroblast’s count in scratch zone from Time 0 h. to Time 18 h. (**D**) Migrating keratinocytes decreased the scratch width and measured in micrometers (μm) at Time 30 h. (**E**) Migrated keratinocyte’s count in the scratch zone from Time 0 h to Time 30 h. (**C**,**F**) Migration assay: immunofluorescence staining with DAPI and Phalloidin. The scale bar shown in micrometers (μm) at the bottom of each image. Graphical results were presented as mean ± Standard Deviation (SD), (* *p* < 0.05, ** *p* < 0.01), *n* = 5.

Fibroblast and keratinocyte migration during skin healing is essential for wound closure [26]. The migratory potential of normal human fibroblasts and neonatal keratinocytes exposed to serotonin, was measured through scratch assay (Figure 2). Experimental observations from migration assay revealed that serotonin treatment significantly increased the number of migrating cells from scratch margin into the scratch zone, resulting in reduction of scratch width in fibroblasts (482 ± 36.10 μm) (Figure 2A – C) and neonatal keratinocytes cultures (406 ± 242.6 μm) (Figure 2D – F) as compared to control (750 ± 96.1 μm), whereas both fluoxetine and ketanserin inhibited fibroblasts and keratinocytes migration, resulted in lack of scratch closure.

### 2.3. Inhibition of Serotonin Pathway Disturbs Skin Wound Healing in Post-Thermal Injury (In Vivo Model)

**Figure 3:**
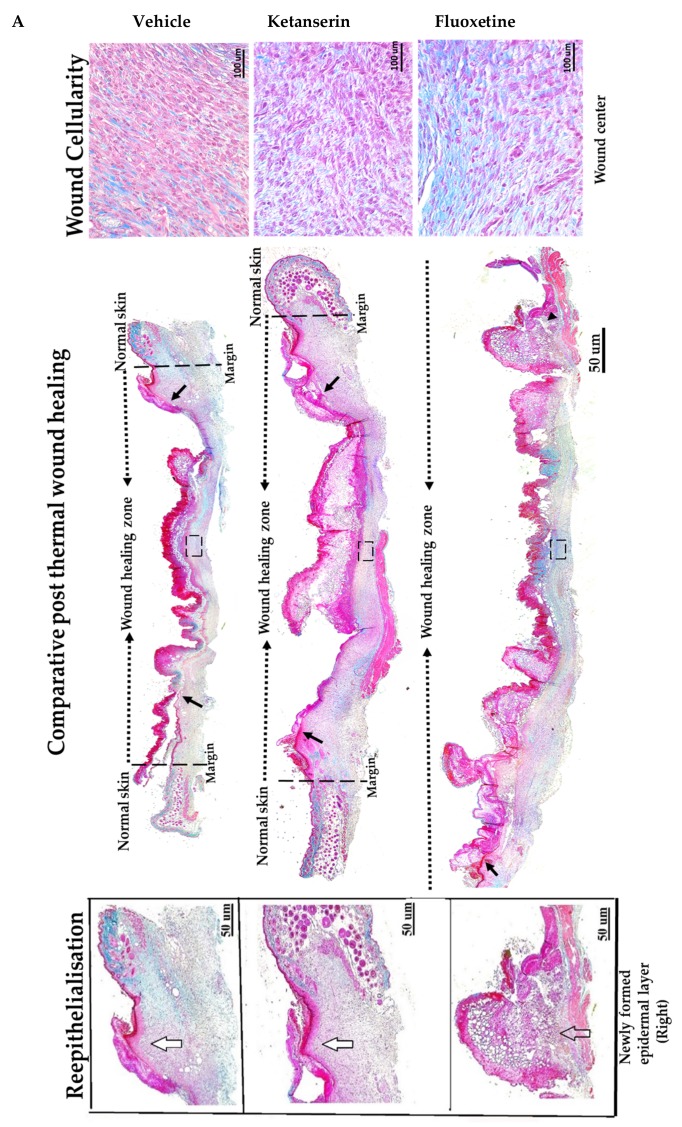

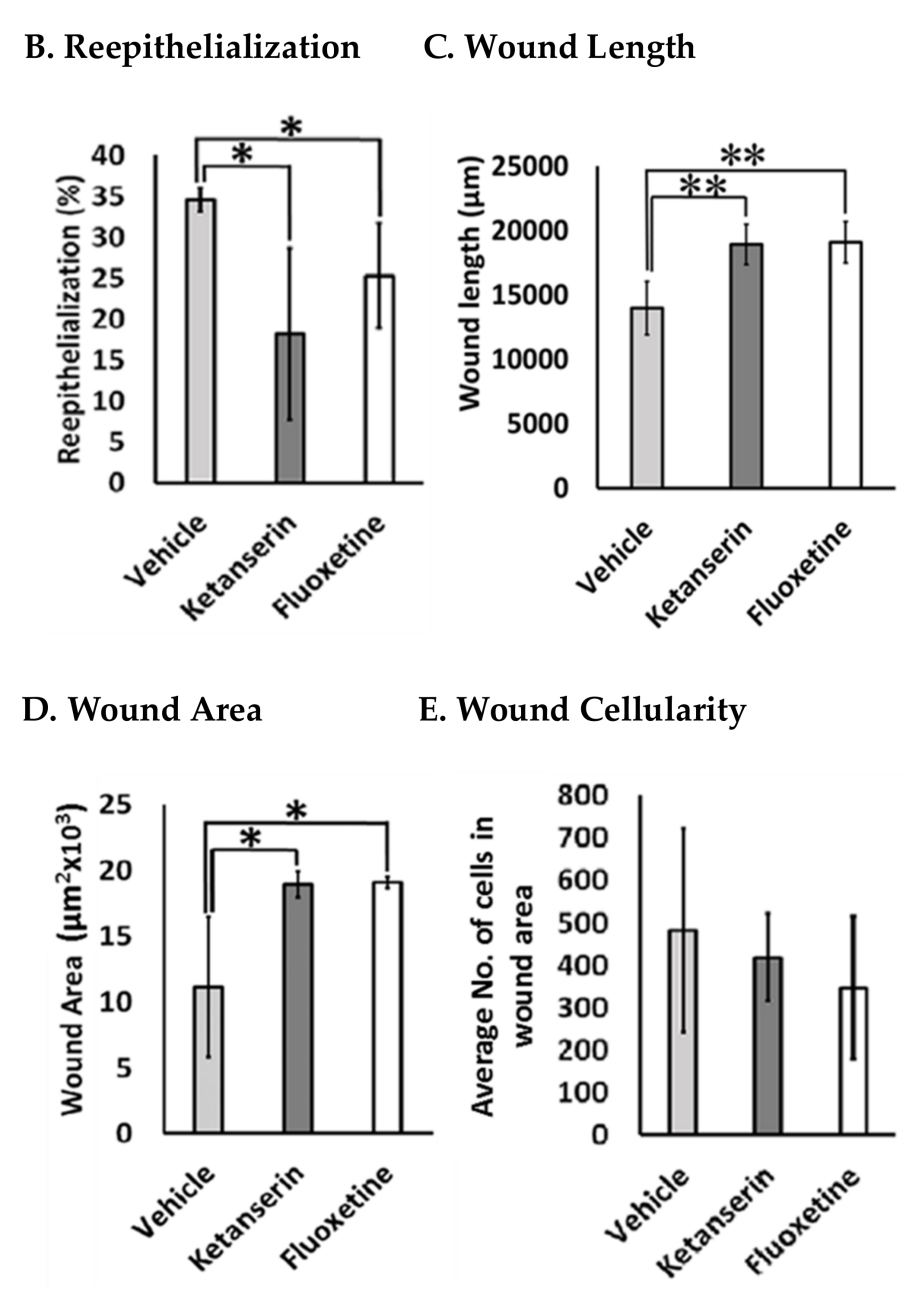
Comparative post thermal skin wound healing in vehicle, ketanserin, and fluoxetine treated groups. (**A**) Masson’s trichrome staining of two weeks’ post burned skin wound cross-sections (image center). Burn wound area characterized with reddish scab (present over wound), distinguishes it from normal skin. Epidermis stained in red and dermal collagen fibers are stained in blue. Reepithelialization: wound images show new epidermal edge (marked with black arrows), formed over left and right wound margins (dotted black line), and progressing towards the wound center (dotted black arrow head line). Image scale: 50 μm. New epidermal layer zoomed at right margins (white arrows mark the newly formed thick layer of epidermis and transparent arrow indicate poorly formed epidermis) (image left). Wound dermal area characterized with granulation tissue. Images show wound cellularity at wound dermal region (dotted black box showed selected area from each image) (image right). Image scale: 100 μm. (**B**) Graphical results show comparative reepithelialization (%), (**C**) wound length (μm) (**D**) wound area (μm^2^ × 10^3^) and (**E**) wound cellularity (average number of cells in wound healing zone) among treatments after two weeks’ post thermal injury. Results were presented as mean ± Standard Deviation (SD), (* *p* < 0.05, ** *p* < 0.01), *n* = 5.

In vivo thermal injury model was employed to analyze the role of serotonin pathway in skin healing (Figure 3). The wound healing process was monitored for two weeks after inducing thermal injury, till harvest. Masson’s trichrome staining was applied on wounded skin sections to observe the possible histological differences between treatment groups. The wound area was characterized by having scab over the wound, underlying newly formed thick epidermal layer and a dermal layer having high fibroblasts count with poorly developed structure as compared to neighboring normal skin sections (on left and right wound margins) (Figure 3A). Results from the control group represent the effects of endogenous serotonin in post burn healing, whereas observations from treatment groups represent the effects on healing after blocking endogenous serotonin with fluoxetine and ketanserin treatments.

Experimental observations from Masson’s trichrome staining of skin wound sections revealed that the control group showed significantly small and better healed wounds as compared to the fluoxetine and ketanserin treatments. Epidermal layer of wound sections from the control group showed total 34.5 ± 1.7% reepithelialization (Figure 3B) by newly formed epithelial cover (on both sides), over wound dermal area, as compared to fluoxetine (25.3 ± 7.3%, *p* < 0.04) and ketanserin (18.2 ± 11.9%, *p* < 0.036) treatments. These results indicate that both drugs significantly suppressed the reepithelialization process, and resulted in completely exposed wounds. Dermal wound length was measured between two normal skin margins and results revealed significantly smaller wounds in control group as compared to ketanserin and fluoxetine treated wounds (20,716.9 ± 1786.6 μm > 19,100.8 ± 1823.6 μm > 13,991.8 ± 2358.7 μm) (Figure 3C). Later, wound area was also measured, which showed similar out comes i.e. significantly bigger wound size in both treated groups as compared to control (18.9 × 10^3^ ± 1.1 μm and 19.078 × 10^3^ ± 0.48 μm > 11.11 × 10^3^ ± 6.1 μm, respectively) (Figure 3D). During the healing process, formation of granulation tissue in the dermal region, is parallel to the reepithelialization. Masson’s trichrome staining showed that granulation tissue (comprising of cellular components) was covering the whole wound dermal area in the control group (Figure 3A). Whereas, both ketanserin and fluoxetine treatments resulted in dermal cell count reduction (average no. of cells in wound area = 417.9 ± 11.9 and 346.5 ± 128.8 respectively, non-significant), and exhibited poorly formed granulation tissue as compared to the control group (482 ± 273) (Figure 3E). These experimental observations revealed that blocking the endogenous serotonin with ketanserin and fluoxetine treatments disturbs the formation of granulation tissue, whereas improved cellularity in wound dermal area of the control group showed the effects endogenous serotonin stimulation (Figure 3A, D). Collectively, results from Masson’s trichrome staining shows that ketanserin and fluoxetine treatments disturbed the reepithelialization and wound cellularity, ultimately resulted in bigger wound size at two-week post-thermal injury. These observations suggest the positive role of endogenous serotonin during wound healing.

### 2.4. Inhibition of Serotonin Pathway Dysregulates the Remodeling Phase of Skin Healing (In Vivo Model)

**Figure 4:**
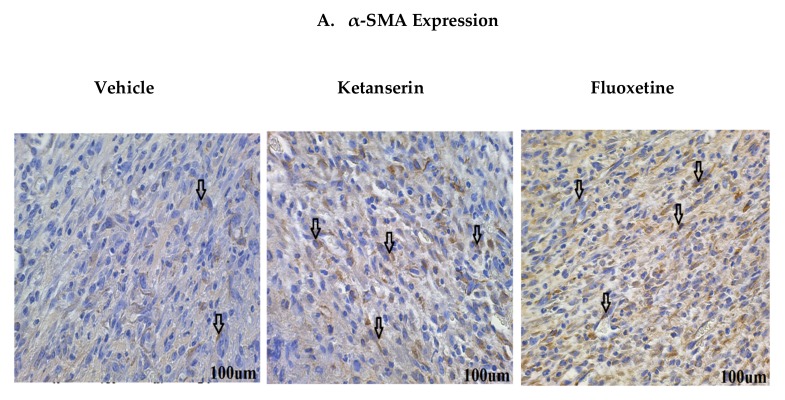

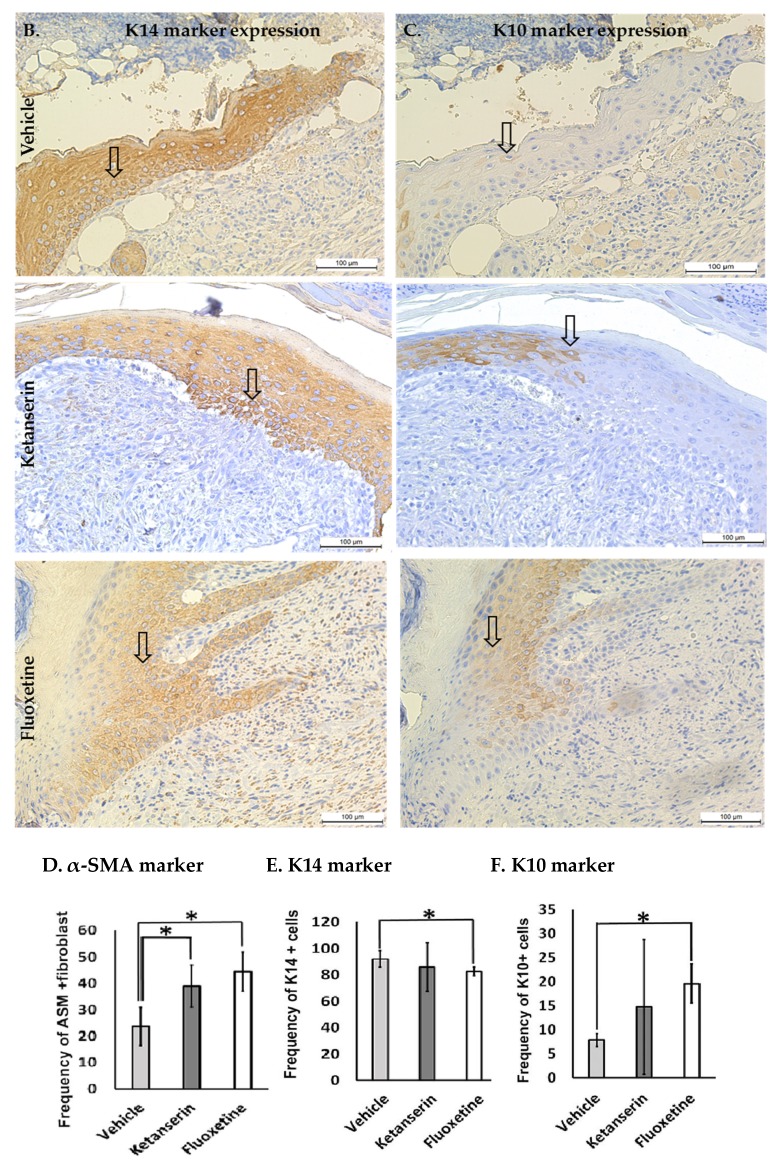
Comparative expression of dermal myofibroblasts alpha smooth muscle actin (α-SMA), epidermal K14 and K10 markers during skin wound healing in vehicle, ketanserin and fluoxetine treatment groups. Two weeks post thermal skin wound sections were harvested and immunostained for these markers. (**A**) Expression of α-SMA in wound dermal fibroblasts (arrow indicate α-SMA + ve cells); (**B**) Expression of K14 and (**C**) K10 in newly formed epidermal layers over the wound area (arrow indicate K14 and K10 + ve keratinocytes in separate images). Image scale bar: 100 μm. Graphical results show comparative (**D**) α-SMA (**E**) K14 and (**F**) K10 expression among treatments after two weeks’ post thermal injury. Results were presented as mean ± Standard Deviation (SD), (* *p* < 0.05), *n* = 5.

Normally, in acute wounds, myofibroblasts are only expressed transiently [27]. The proliferation phase is marked by extensive myofibroblast expression that are involved in collagen deposition, as well as wound closure. Alpha smooth muscle actin (α-SMA) is expressed by myofibroblasts during wound healing and is considered to be a wound maturation marker [28]. In order to find out the effects of serotonergic drugs on myofibroblasts expression, dermal fibroblasts were immunostained for α-SMA marker (Figure 4A). Experimental results showed that fluoxetine and ketanserin treatments significantly enhanced the α-SMA expression (44.36 ± 8.5, *p* < 0.005 and 38.98 ± 9.1, *p* < 0.023, respectively) as compared to the control group, which showed a lower α-SMA count (23.68 ± 8.2) (Figure 4D). The elevated myofibroblast expression in wound dermal area after two weeks post-burn injury suggests SSRI-mediated peripheral serotonin impairment. Interestingly, lower α-SMA expression in control group correlates with a smaller wound size. Additionally, in ketanserin treated group 5HTR2a antagonism suggests that there might be a dysregulated proliferation and remodeling in burn wounds resulting in poor wound healing.

### 2.5. Fluoxetine Enhances Keratinocyte Differentiation at the Edge of Healing Skin (In Vivo Model)

Epidermal keratinocytes play an essential role in shaping the outcome of skin healing [29]. To assess the effects of inhibiting the serotonergic pathway on the differentiation state of keratinocytes during skin healing, keratin 14 (K14) and keratin 10 (K10) expressions were assessed by immunostaining (Figure 4B, C). K10 expression is a marker of terminally differentiating keratinocytes in their post-mitotic phase [30], whereas K14 expression is associated with keratinocyte proliferation [31]. Observations showed that fluoxetine treatment resulted in significantly low K14 expression (frequency of K14 + ve cells, 82.56 ± 3.6, *p* < 0.023) (Figure 4E) in keratinocytes as compared to the control group (frequency of K14 + ve cells, 91.98 ± 7.1). In contrast, fluoxetine treatment showed increased expression of K10 (frequency of K10 + ve cells, 19.57 ± 11.01, *p* < 0.038) (Figure 4F) as compared to the control group (frequency of K10 + ve cells, 7.87 ± 1.4), whereas for ketanserin treated groups K10 and K14 expressions were not different from control.

## 3. Discussion

It has been previously suggested that serotonin may have an important role in promoting post-injury homeostasis and skin healing [32]. Serotonin’s ability to induce fibrosis in the skin was documented for the first time in 1958 by MacDonald, et al.; however, this study explored the effects of serotonin administration on uninjured normal skin [20]. But, its role in cellular proliferation in the context of cutaneous wounds has not been explored. We decided to study the effects of SSRIs because they specifically inhibit serotonin transporter (SERT) and deplete platelets’ intracellular stores of serotonin [33], thereby impairing platelet-induced serotonergic signaling [34]. Whereas, 5HTR2 signaling has also been implicated previously, to study the fibro-proliferative effects of serotonin in cardiac, hepatic, renal and pulmonary tissues [16,35,36]. However, the effects of SSRI and 5HTR2A antagonist have not been previously studied in the context of cutaneous burn injuries. Therefore, by using these drugs, this study evaluates the role of endogenous serotonin stimulation and inhibition in term of downstream serotonin signaling in fibroblast and keratinocyte’s viability, apoptosis, proliferation and migration during skin healing after thermal injury.

Previous investigations have shown that apart from tissue necrosis, apoptosis also plays an important role in wound expansion in burns [37,38]. Subsequently, it has been suggested that countering apoptosis might have some therapeutic implication in controlling burn wound progression [38]. Therefore, we investigated the effects of serotonin on apoptosis in the context of burn wounds. Our in vitro culture results show that serotonin treatment significantly enhanced cell survival in fibroblasts by inducing anti-apoptotic effects. Moreover, serotonin administration enhanced cell proliferation and migration in dermal fibroblasts and keratinocytes as well. Whereas, ketanserin (5HTR2A inhibitor) treatment reduced the cell viability, proliferation and migration in dermal fibroblasts and keratinocytes. Additionally, our in vivo experiments, further provide the substantial support to these results. In vivo experiments revealed that following acute cutaneous thermal injury, ketanserin treatment results in bigger wound area, and significantly reduced reepithelialization. Experimental observations from the ketanserin treatment group in our wound healing experiments correlate with previous studies which report that 5HT2A-antagonism has been associated with anti-inflammatory response of macrophages, via suppression of a key pro-inflammatory transcription factor NF-κB and its downstream inflammatory cytokines including IL-12 and TNF-α from macrophages [24,39]. However, in the context of acute wounds, adequate transient inflammatory response is necessary and such immunosuppression leads to delayed wound closure, reduced fibroblast proliferation, and reepithelialisation [25]. Similar out comes have been observed in our experiments. Therefore, it is possible that the increased wound area and impaired epithelialization following ketanserin treatment is due to the impairment of the inflammatory response. Our results showing suppression of fibroblast and keratinocyte proliferation with ketanserin treatment also support previous studies indicating that ketanserin reduces fibroblast proliferation and induce fibrosis in the liver [40], whereas it is also documented that activation of 5HTR2A signaling promotes fibrosis in various other tissues [16,33,34].

Previously it has been reported that ketanserin had no significant effect on wound healing during acute cutaneous injury [41]; similarly, our results also revealed that ketanserin impairs burn wound healing as well. Although previous study examined the surgical incision injuries instead of burns and utilized topical 2% ketanserin gel instead of our systemic ketanserin treatment [41]. Systemic ketanserin application would have wide-spread effects by antagonizing all 5HTR2A expressing cells including platelets and lymphocytes [42,43]. Thus, retrospectively, topical ketanserin treatments are advantageous for studying effects of ketanserin on local wound environment. Regardless, both studies agree that neither systemic ketanserin treatment nor topical ketanserin treatment has been found advantageous for acute wound healing.

Like ketanserin, fluoxetine administration in mice with cutaneous thermal injuries also showed bigger wound size and a significant reduction in reepithelialisation. Systemic administration of SSRI has been previously reported to disrupt platelet intracellular serotonin stores by inhibiting SERT. Consequently, the lack of platelet-mediated serotonin release, results in impaired hemostasis [44] and systemic immunosuppression, as exemplified by fluoxetine-mediated T-cell immunosuppression in murine graft-versus-host disease [45]. Since hemostasis and immune response are vital components of wound healing, SSRIs have been suspected to have deleterious effects on wound healing. Our results confirm these suspicions and highlight the degree of impairment in the context of burn wounds, in fluoxetine treated group. In agreement with our conclusions, it has been previously documented that following punch-biopsy wounds in mice skin, mice that were TPH-1 deficient and completely lacked peripheral serotonin and those that were systemically treated with SSRI also resulted in impaired wound healing [46]. In contrast, one study has reported fluoxetine enhanced cutaneous wound healing in chronically stressed Wistar rats [47]. Although it may be possible that providing adequate psychiatric anxiolytic treatment in individuals, predisposed to anxiety may have some benefit to wound healing due to behavioral changes, our results combined with previous studies do not suggest that systemic SSRIs are beneficial for wound healing.

Interestingly, fluoxetine also showed decreased fibroblast and keratinocyte proliferation in in vitro culture conditions. It is likely that SERT inhibition may specifically affect fibroblast and keratinocyte proliferation and migration. Previous studies in CNS have shown that although SERT inhibition initially results in increased extracellular serotonin concentrations due to decreased re-uptake, this eventually leads to down regulation of several 5HTR receptor subtypes including 5HTR1A [48]. This down regulation of serotonin receptors might paradoxically impair serotonin signaling despite having excess extracellular serotonin. Our results show that in vivo fluoxetine treatment enhanced K10 expression and suppresses K14 expression. Looking at the data holistically and considering trichrome staining showing fluoxetine administration caused reduced re-epithelialization and keratinocyte migration, which explains the fact that fluoxetine may be inducing pre-mature terminal differentiation of keratinocytes, resulting in impaired migration and poor closure of the wound. The enhancement of keratinocyte terminal differential leading to impaired wound healing is a known concept and has been demonstrated previously in other agents including glucocorticoids [49]. These effects were not observed for ketanserin, suggesting that 5HTR2A inhibition does not affect the expression of K10 and K14, and may affect wound closure through different mechanisms.

Moreover, we observed that, although ketanserin and fluoxetine administration decreased the fibroblast proliferation in post-burn mice, but they also resulted in increased α-SMA expression, which may suggests increased fibroblast to myofibroblast trans-differentiation [50]. In normal acute wound healing process, myofibroblasts undergo apoptosis after re-epithelialization and wound closure [51]. Various cellular and matrix-based cytokines and growth factors including connective tissue growth factor (CTGF), platelet-derived growth factor (PDGF), TGF-β, matrix hyaluronic fragments and physical factors such as mechanical stress are involved in maintaining the myofibroblast pool [52]. Therefore it is likely to infer that, the perpetuation of myofibroblasts in ketanserin and fluoxetine-treated wounds might be due to the impaired re-epithelialization and failure of wound closure. Furthermore, interactions of fibroblasts with keratinocytes are also known to promote re-epithelialization; however, myofibroblasts have a limited role in re-epithelialization but play a greater role extracellular matrix secretion and wound closure [53]. Having low fibroblasts and increased proportion of myofibroblasts supports our findings of impaired re-epithelialization with ketanserin and fluoxetine administration. Our results conflict with a previous study that suggests systemic ketanserin administration causes down regulation of α-SMA expression in portal fibroblasts pre-treated with serotonin [40]. However, this study evaluated the mRNA and protein expression of cultured portal fibroblasts, while our observation was based on quantifying α-SMA-positive cells using skin tissue immunostaining. Also, burn wounds have a predisposition to have high and prolonged myofibroblast expression [22,54], which means that the biliary environment may not be comparable with the burn microenvironment.

Since burn injuries are also characterized by dysregulated fibrosis [22], given our findings of decreased fibroblast proliferation upon ketanserin and fluoxetine administration, it is natural to speculate whether they might have some relevance to the hypertrophic scarring [54]. However, since myofibroblasts are also known to deposit collagen, leading to the hypertrophic scarring [25,55], it is likely that they may not prove efficacious in this regard. Moreover, topical administration of SSRIs could potentially worsen hypertrophic scarring. Theoretically, with the low systemic absorption of topical SSRIs, platelet function should remain intact, but the local extracellular serotonin might increase. This can hypothetically occur by topical SSRI antagonizing serotonin re-uptake by local keratinocytes, fibroblasts, and leukocytes, all of which express SERT [42]. As serotonin up regulation is already associated with fibrosis [56], adding it to a microenvironment like burn wounds that already has the propensity for a prolonged inflammatory phase and hypertrophic scarring [57] may not be beneficial to prevent scarring. However, if one’s objective is to rapidly close the wounds, topical serotonin could enhance local serotonergic effects leading to a more profound inflammatory phase and a rapid wound healing process.

The present study has advanced the understanding of the role of peripheral serotonin in cutaneous wound healing process during burn injuries. As SSRIs inhibit platelet-mediated serotonergic effects and ketanserin inhibits 5HTR2A-signalling, the impaired wound healing upon administration of these agents suggest the positive role of endogenous peripheral serotonin for the reepithelialization process during wound healing. Since endogenous serotonergic signaling appears to enhance proliferation and migration of keratinocytes and fibroblasts, therefore serotonin or 5HTR agonists could be a potential candidate for enhancing skin healing in burn patients. It is important to mention that the in vitro studies particularly scratch assay is dependent not only on the level of cell migration but also the proliferative state of the cells. Considering that serotonin affects proliferation, this is a limitation to our methods. The in vivo study also has a few limitations, as we realize that there is a need to harvest and analyze all four stages of wound healing.

## 4. Materials and Methods

### 4.1. Skin Tissue Sample

The skin samples were collected from dermatologically healthy patients (undergoing surgical procedures) and burn patients, with donor’s consent. Samples were collected at the Ross Tilley Burn Centre, Sunnybrook Health Sciences Centre, Department of Plastic Surgery, University of Toronto, Toronto, Canada. The dermis of these skin tissue samples, was used to culture primary fibroblasts, and snap frozen at −80 °C for preservation.

### 4.2. Cell Culture

Normal human primary skin fibroblasts were obtained from normal human skin tissue samples and dissected into 2 to 4 mm sized small pieces (explant). Explant further sub-cultured in culture dishes containing fibroblast culture medium (high glucose Dulbecco’s modified Eagle’s medium supplemented with 10% fetal bovine serum and 1% antibiotic/antimycotic solution) at humidified atmosphere (37 °C and 5% carbon dioxide) for one week. The primary culture at 70% confluency level was subjected to trypsinization (0.05% trypsin). Fibroblasts further sub-cultured at cell density of 4500 cells/cm^2^ in flasks (cell culture flasks 75 cm^2^, Corning^®^, Corning‎, NY, USA). Normal neonatal epidermal Keratinocytes (HEKn) were purchased from Life technologies (Gibco™, Burlington, ON, Canada) and cultured in EpiLife^®^ Medium, with 60 μM calcium (Gibco™, Burlington, ON, Canada) and Human Keratinocyte Growth Supplement (HKGS) [58].

### 4.3. In Vitro Serotonin Treatment

#### 4.3.1. Drugs

Serotonin (Serotonin Creatinine Sulfate Monohydrate), Ketanserin (Ketanserin (+)-tartrate salt). Fluoxetine (Fluoxetine hydrochloride solid) and Dimethyl Sulfoxide (DMSO) were purchased from Sigma-Aldrich (Oakville, ON, Canada). Experimental drug concentrations were selected based on previous studies [59,60,61] and all three drugs were optimized for both keratinocyte and fibroblast cultures. The dose selection criteria were based on the concentrations of serotonin that favor higher proliferation and decreased apoptosis, both in keratinocytes and fibroblasts in vitro cultures. Therefore, experimental evaluation revealed optimized dose combinations as serotonin 10^−4^ M, ketanserin 10^−9^ M and fluoxetine 10^−8^ M. The optimized drug dose was used throughout the experimental analysis.

#### 4.3.2. Apoptosis Assay

Effects of serotonin was evaluated on cell apoptosis by quantification of caspase-3/7 activities in human fibroblasts and neonatal keratinocytes (HEKn). Caspase activity was determined by a commercially available luminogenic caspase-3/7 substrate assay kit method (Caspase-Glo^®^ 3/7 Assay, Promega, Madison, WI, USA). Briefly, cells were seeded in 96-well plates at a density of 5000 cells/ well in serum-free RPMI media and permitted to adhere for 24 h. Old media was replaced and cells were challenged with fresh, serum-free respective media containing the tested drugs at the specified concentrations: serotonin (10^−4^ M), ketanserin (10^−9^ M) and fluoxetine (10^−8^ M). Negative control: cells were incubated with the vehicle “DMSO”. All treatment groups were incubated for 12 h and then caspase-3/7 activity was performed. Relative fluorescence (RFU) over a 1 s interval was measured in a microplate reader and observations were recorded [58].

#### 4.3.3. Viability Assay

Human normal skin fibroblasts and normal neonatal Keratinocytes (HEKn) cultures were seeded in 96-well culture plates at 5000 cells per well in respected serum free media for viability assay. Cultures were incubated at 37 °C in a humidified atmosphere (5% carbon dioxide), until 50% confluent, followed by 24 h of serum free media replaced by a fresh media containing drugs serotonin (10^−4^ M), ketanserin (10^−9^ M) and fluoxetine (10^−8^ M) or DMSO as a vehicle control. Viability assay performed by using CellTiter-Glo^®^ Luminescent cell viability assay kit (Promega, Madison, WI, USA) and absorbance was measured at 550 nm on a microplate reader. Results were analyzed and presented as Relative Luminescent Unit (RLU) [62].

#### 4.3.4. Proliferation Assay

Human normal skin neonatal keratinocytes (HEKn) and fibroblasts were cultured (cell density of 6000 cells/cm^2^) in cell culture slides (eight-chamber glass slides) separately until 50–60% confluent. Then culture media was replaced by a fresh medium containing test drugs. Bromodeoxyuridine, BrdU (1:200) was incorporated in each chamber at the same time and kept for incubation (24 h) [63]. Immunofluorescence analysis: Cultures were washed with PBS (phosphate-buffered saline) and fixed with Paraformaldehyde 4% (Alfa Aesar, Karlsruhe, Germany) for 15 min. Cells were washed with phosphate-buffered saline and subjected for permeabilization with PBST (containing 0.5% Triton X-100 solution and PBS) for 10 min. After wash, fixed cells were subjected to blocking (30 min) with Bovine serum albumin (1% BSA) in PBST. The primary monoclonal antibody, mBrdU, ratio 1:200 (cell signaling, Danvers, MA, USA) was used for overnight incubation (4 °C). Cells were washed with PBS. The secondary antibody anti-mouse, Alexa Fluor-488, ratio 1:500 (Life Technologies, Burlington, ON, Canada) was added on the slides and kept for incubation in dark (room temperature, 1 h). After incubation slides were washed with PBS and subjected for mounting with medium Vectashield, containing (4, 6-diamidino-2 phenylindole) DAPI (Vector Laboratories, Burlingame, CA, USA). After staining, cells were observed and exposed for photographs (20× magnification) by using Apotome Axiovert fluorescent system Zeiss (Göttingen, Germany). Results were presented in term of mean frequency of BrdU + ve cells with 95% confidence intervals [64].

#### 4.3.5. Cell Migration Study (Scratch Wound Assay)

To determine the effects of serotonin and serotonergic modulating agents on cell migration, human normal skin neonatal keratinocytes (HEKn) and fibroblasts were cultured (20,000 cells each) in glass culture slides (Thermo Scientific, Lab-Tek chamber slides, Burlington, ON, Canada) for 24 h. Scratches were made by using 1000 μL pipette tip (two scratches per well). Following with PBS wash old media was replaced with fresh media containing test drugs and incubated for 24 h [62]. Immunofluorescence analysis: 4% Paraformaldehyde was used to fix the cultured cells. First Phalloidin antibody (1:30) conjugated to fluorescein isothiocyanate (Invitrogen, Burlington, ON, Canada) was added into cell culture and incubated for 1 h in blocking solution. Cells washed with PBS and subjected for mounting with medium Vectashield, containing DAPI and processed for imaging. Imaging (5× magnification) was carried out by using a Laser scanning microscope (META 510 confocal microscope, Zeiss, Göttingen, Germany) and quantified by the ImageJ software program, National Institutes of Health (Bethesda, MD, USA) [64].

### 4.4. In Vivo Wound Healing Model

#### 4.4.1. Animal

C57BL/6 mice were purchased from Jackson Laboratories (Bar Harbor, ME, USA). The study approved (AUP: 17-503, expiry date: 14 June 2018) and followed all guidelines of Animal Policy and Welfare Committee at Sunnybrook Research Institute and the University of Toronto, Toronto, ON, Canada.

#### 4.4.2. In Vivo Thermal Injury Procedure

Animals (male C57BL/6 mice, body weight 25 g, 6 weeks old) were divided into three experimental groups: (1) ketanserin; (2) fluoxetine; and (3) control group. All groups were subjected to a scald burn animal protocol (AUP: 17-503) [65]. Briefly, the mice were anesthetized with isoflurane (2–3%) and body weights were recorded. Dorsal surface of the mouse was shaved. Buprenorphine was administered by intraperitoneal injection. Ringer’s lactate solution (1 mL, intraperitoneal injection) was administered to resuscitate the mice. After anesthesia the mouse was positioned in a mold (exposing 20% TBSA; total body surface area, along with whole dorsal spine surface). The mold containing mice was immersed in hot water (98 °C water, 10 s) to produce scald burn, and then put it back into experimental cages. Mice were monitored until they were fully awake and shifted back into the animal room. Sham treatment only received anesthesia and Ringer’s lactate solution induced resuscitation. Ketanserin, fluoxetine and vehicle groups received daily systemic treatment via intraperitoneal administration (10 mg/Kg of body weight) for the respective drugs, up to two weeks post-burn. Mice were sacrificed on day 14 and biopsies were taken from wound sites and normal skin area, for comparison. Skin wound samples were processed for respective skin wound histology [66].

### 4.5. Skin Histology

Skin tissue specimens from each group were subjected to fixation. Histological assessment was carried out on skin sections derived from the widest part of the wound (wound center). Thus, most completely disrupted part of the wound was considered for healing assessment. By implementing this strategy we were able to assess distinct changes in the wound-healing process and ensure reproducibility. Tissue specimens were fixed in 10% buffered formalin (overnight at room temperature), preserved in 70% ethanol and embedded in paraffin. Tissue specimens were cut simultaneously, at different sites, the center or midline and both sides, eliciting a cross-section through the whole wound and satellite area. A serial section of the scar or wound was performed. The largest wound diameter or central wound section was stained for trichrome staining [37].

### 4.6. Trichrome Staining

Paraffin-embedded slides were deparaffinized with citrosol, followed by rehydration through 100%, 95%, 70% and 50% ethanol to water. Slides were placed in Bouin’s solution (26367-01; EMS, Hatfield, PA, USA) overnight at room temperature and washed. Hematoxylin stain (HHS16; Sigma, Saint Louis, MO, USA) and Biebrich scarlet-acid fuchsin solution were applied sequentially for 10 min. After each stain slides were washed. Next, slides were differentiated in phosphomolybdic–tungstic acid for 15 min, and transferred to aniline blue for 5 min. All slides were rinsed properly and differentiated in 1% acetic acid for 2 min. Slides were dehydrated through 95% ethanol and absolute ethanol followed by clearing in citrosol. Slides were mounted with xylene-based liquid mounting media (Triangle Biomedical Sciences, Durham, NC, USA). All Trichrome reagents were purchased from EMS (Hatfield, PA, USA). After completing all staining steps, slides were subjected to photography by using a light microscope (Zeiss Axiovert 200; 5× and 40× magnification) and subsequent quantification analysis for respective study [67].

### 4.7. Immunohistochemistry

Paraffin-embedded skin tissue slides were subjected to immunohistochemistry staining procedure. First, xylene was added to deparaffinize and followed by different stages of rehydration according to the protocol. Antigen decloaker, 1× (Biocare Medical, Pacheco, CA, USA) was poured into the slides and kept in decloaking chamber (preheated for 4 min at 110 °C). Washing buffer (0.05 M Tris–HCl, 0.15 M NaCl and 0.05% Tween 20) was used to wash the slides. Primary monoclonal mouse antibody anti-ms α-ASM, 1:400 (eBiosciences, Burlington, ON, Canada), rabbit anti-K14 and K10, 1:400 (Covance, Princeton, NJ, USA) prepared in PBS, added on slides and kept for incubation (one hour, room temperature). First, mouse probe MACH3 (Biocare Medical, Pacheco, CA, USA) was added on slides (incubation, 15 min), then mouse or rabbit MACH3 polymer Horseradish peroxidase was added and again incubated for 15 min. Chromogen betazoid diaminobenzidine kit (Biocare Medical, Pacheco, CA, USA) was added and noticed until stained brown. The reaction was terminated by washing under running water. Slides were stained with Hematoxylin for 30 s and differentiated in acid alcohol 1.5% and 0.1% sodium bicarbonate for 10 s. Slides were dehydrated by dipping in 95% ethanol, absolute ethanol, and citrosol. Stained slides were mounted with xylene. Images were obtained by using a light microscope (Zeiss Axiovert 200, magnification at 10× and 40×) and quantified [68].

### 4.8. Statistical Evaluation

Experimental validity was established by performing all in vitro assays five times and in vivo experiment three times for each treatment group. All results were presented as mean ± standard deviation (SD), 95% confidence interval after applying student’s *t*-test by using Microsoft Excel version 8. *P* value ≤ 0.01 and 0.05 were considered as significant. We analyzed the data again by using two-way ANOVA and Tukey post-hoc tests at significance level of *p* ≤ 0.05.

## 5. Conclusions

Serotonin has an important role in wound healing in the context of burn injuries. Serotonin promoted cellular viability, proliferation and migration of both fibroblasts and neonatal keratinocytes (in vitro), whereas inhibition of the 5HTR2A receptor by ketanserin resulted in opposite effects. Moreover, ketanserin administration results in bigger wound size and decreased re-epithelialization in post-burn mice skin. Similarly, administration of fluoxetine (a selective serotonin reuptake inhibitor), also dysregulates wound healing. Collectively, these data show that endogenous serotonin enhances proliferation and migration of keratinocytes and fibroblasts, necessary for proper wound healing. Therefore, serotonin or 5HTR agonists can be a potential candidate for enhancing skin healing in burn patients. Further experiments are warranted to investigate the role of other serotonin receptors to enhance our understanding of the serotonergic system’s role in wound healing.
